# Why partisans feel hated: Distinct static and dynamic relationships with animosity meta-perceptions

**DOI:** 10.1093/pnasnexus/pgae324

**Published:** 2024-10-15

**Authors:** Jeffrey Lees, Mina Cikara, James N Druckman

**Affiliations:** Andlinger Center for Energy and the Environment, Princeton University, Princeton, NJ 08540, USA; Department of Human Resource Management and Organizational Behavior, University of Groningen, 9747 AE Groningen, The Netherlands; Department of Psychology, Harvard University, Cambridge, MA 02138, USA; Department of Political Science, University of Rochester, Rochester, NY 14627, USA

**Keywords:** meta-perceptions, elections, social media, intergroup conflict, polarization

## Abstract

Partisans hold inaccurate perceptions of the other side. What drives these inaccuracies? We address this question with a focus on partisan animosity meta-perceptions (i.e. how much a partisan believes opposing partisans hate them). We argue that predictors can relate to meta-perceptions statically (e.g. at a specific point in time, do partisans who post more about politics on social media differ in their meta-perceptions relative to partisans who post less?) or dynamically (e.g. does a partisan who increases their social media political posting between two defined time points change their meta-perceptions accordingly?). Using panel data from the 2020 US presidential election, we find variables display distinct static and dynamic relationships with meta-perceptions. Notably, between individuals, posting online exhibits no (static) relationship with meta-perceptions, while within individuals, those who increased their postings over time (dynamically) became more accurate. The results make clear that overly general statements about meta-perceptions and their predictors, including social media activity, are bound to be wrong. How meta-perceptions relate to other factors often depends on contextual circumstances at a given time.

**Significance Statement**

Why do partisans misperceive the opposing party? The answer depends on whether the focus is on static relationships at a single point in time, or dynamic relationships that involve change within individuals, over time. For instance, during the 2020 US presidential election, perceptions of how hateful the other party is did not differ between those who frequently posted about politics on social media and those who did not. Yet, perceptions did change among those who increased their political posting from pre- to postelection. They came to believe the other party was *less* hateful. Predictors of polarization, particularly beliefs about the other party, can operate differently in static versus dynamic settings. Moreover, the influence of social media remains far from clear.

## Introduction

A defining feature of 21st century American politics is polarization. One of the most dramatic manifestations concerns the tendency for members of political parties to perceive their opponents as much more hateful, spiteful, demographically stereotypical, undemocratic, and violent than they actually are (1–6). These polarized (mis-)beliefs fray interpersonal relationships and democratic governance since people are averse to engaging or compromising with such noxious others (7–9). Indeed, Dimant (10) shows that pessimistic expectations about the opposition's cooperation breeds intergroup conflict.

Despite a growing literature on interventions that correct partisans’ misperceptions (5, 10–13), scant work explores the nature and origins of inaccurate misperceptions in the first place. What predicts exaggerated or inaccurate beliefs? Here, we focus on partisan animosity meta-perceptions: how much animus a partisan (e.g. Democrat) believes members of the other party (Republicans) hold toward their own party (Democratic Party) (14). We begin by providing a framework for studying misperceptions (including exaggerated meta-perceptions). We make the case for quantifying both static (between individuals) and dynamic (within individual) relationships between perceptions and other variables. The same variable (e.g. posting about politics online) can exhibit different static (e.g. null or negative) and dynamic relationships (e.g. positive) with perceptions of the other party. We then describe the data we analyze, from the 2020 US presidential election, as well as the methods we employ. We present our descriptive results and conclude with advice for future work, emphasizing the importance of accounting for the static and dynamic inputs to beliefs and perceptions. Doing so is crucial since static relationships (as commonly studied at a moment in time) might not be the same as dynamic ones (as studied longitudinally, over time).

## A framework for studying static and dynamic relationships

Our framework for studying the relationship between meta-perceptions and other variables starts from two premises. First, meta-perceptions depend on both features of the individual and features of the context (e.g. (15–17)). For instance, partisan animosity meta-perceptions might depend on both an individual's ideological extremity (e.g. relative to moderates, ideologues may believe the other side hates them more since they exhibit greater disagreement) and the extent of intergroup competition at a given time (e.g. relative to a cooperative context, a competitive situation such as an election could lead individuals to believe the other side hates them more, given the stakes). Second, the relationship between a predictor and meta-perceptions can be static or dynamic.

In the static case, the relationship concerns the value of an explanatory variable (predictor) at a given time, *t*1, and the value of the outcome variable at that same time *t*1. For instance, it might involve exploring whether, relative to those who post little political content on social media, those who post a lot exhibit more exaggerated animosity meta-perceptions. Or, perhaps one studies whether, relative to moderates, extreme ideologues exhibit more inaccurate meta-perceptions. In the dynamic case, the relationship concerns how a change in an explanatory variable for an individual, from *t*1 to *t*2, relates to a change in the outcome variable over that same time period. For instance, one might ask whether an increase (decrease) in an individual's posting political content correlates with more (less) inaccurate animosity meta-perceptions or whether becoming a more (less) extreme ideologue leads to more (less) inaccurate meta-perceptions. Static versus dynamic relationships correspond to studying relationships between individuals or within a given individual over a period of time.

Our two premises have methodological and theoretical implications. In observational data, assessing dynamic relationships provides greater leverage in asserting causality because it controls for time-invariant, unobserved variables (18). For instance, if an individual's change in political posting on social media relates to a change in exaggerated meta-perceptions, one can be confident that enduring variables like personality are held constant. That is not the case for quantifying static relationships in observational data because these are comparisons between individuals^a^. To be clear, in this paper we focus on descriptively documenting variation between static and dynamic relationships rather than acutely identifying causal relationships between our explanatory variables and meta-perceptions (i.e. we do not rigorously rule out possible confounding third variables accounting for the observed relationships even in the within-person analyses).

Static and dynamic relationships produce the same results when certain conditions are met (19, pp. 27–32). One necessary condition of static–dynamic congruency is temporal stability—that is, the relationship does not depend on *when* it is assessed. Consider, for instance, that Lees and Cikara (4) offer suggestive data that individuals exhibit more inaccurate, exaggerated meta-perceptions in competitive contexts than cooperative situations (20). For some variables, this may not be relevant. Extreme ideologues likely have more exaggerated meta-perceptions regardless of the competitive or cooperative nature of the context (i.e. ideological extremity is not moderated by context). For other variables, it may be very relevant; predictors that involve information sharing likely depend on the situation. Posting about politics and receiving feedback right before an election may exacerbate inaccuracies given the likely conflict/disagreement with the other side. If instead, the information environment is relatively cooperative, then increased posting could diminish inaccuracies. The general point aligns with our aforementioned first premise that meta-perceptions reflect not only individual features but also the environment in which they are formed or updated. This coheres with Munger's (21, 22) argument that researchers studying communicative relationships need to consider temporal validity.

This has a theoretical implication. Perhaps, the most significant is that two variables can potentially have different relationships at the static and dynamic levels of analysis, suggesting that disaggregating them can point to meaningfully different hypothetical processes occurring simultaneously. For example, for posting about politics on social media, the static relationship involves comparing meta-perceptions at time *t* between those who frequently post and those who infrequently post. For the dynamic relationship, the question is whether the *change* in how much one posts about politics correlates with that person's meta-perceptions becoming more or less accurate over time. People who frequently post online may generally have more inaccurate partisan animosity meta-perceptions than those who rarely post. Yet, imagine that a high-frequency poster increases the amount they post over two weeks, and during that same period extreme partisans become less active on social media (relative to the preceding weeks; thus, the context changes). In this case, increased postings could correlate with the evolution of a *more accurate* meta-perception because the extreme exemplars are absent. Thus, one could hypothetically find both (ⅰ) that frequent social media posters (relative to low frequency posters) report more inaccurate meta-perceptions (a between-person comparison) and (ⅱ) that an increase in social media postings between two points in time, for those same frequent posters, is associated with a decrease in inaccurate meta-perceptions (a within-person change). These two distinct (example) processes would be obscured analytically without the theoretical and statistical distinction between dynamic and static relationships.

Which variables are more or less likely to exhibit different static and dynamic relationships with animosity meta-perceptions? We expect a continuum ranging from variables that are context- and time-invariant to those that are variant. The invariant part of the continuum includes items that reflect relatively stable qualities. An example would be ideology. At the other end of the continuum are highly malleable measures, sensitive to the context including the content of one's information ecology. An example would be political posting on social media given that what other people post can change substantially over time and vary across political contexts (23). In the middle of the continuum (i.e. moderate likelihood of distinct static and dynamic relationships) are variables that involve information profusion but in a more curated manner (e.g. the composition of one's general social networks). Finally, a distinct point is that, all else constant, we expect stronger static than dynamic relationships. The former are less noisy relationships and have likely crystalized over longer periods of time^b^.

Before turning to our data, we offer two clarifications. First, in the abstract, one could imagine mixes of individual/contextual and static/dynamic relationships; this would require explicitly measuring contextual variations, a point to which we return in the conclusion. Second, while isolating within-person associations provides less biased estimates of (theoretically) causal relationships relative to between-person associations, within-person associations themselves provide no greater inherent capacity for causal inference. Any such capacity for causal inference must come from the nature of the data (e.g. data collected pre/post some event or intervention).

## The 2020 election and partisan animosity meta-perceptions

We evaluate static and dynamic relationships by analyzing a unique two-wave panel dataset of Americans that asked for their partisan animosity meta-perceptions both before and after Election Day in 2020. We focus on party thermometer ratings, which ask respondents to rate the parties on a 0 to 100 scale with lower scores indicating coldness and higher scores indicating warmth. This is the canonical measure used in studies of affective polarization and partisan animosity (24). The panel also asked respondents for their meta-perceptions by querying how much respondents think a typical member of the other party would rate their own party (i.e. a Democrat was asked how a typical Republican would rate Democrats). In our analyses, we use the inverse of animosity or “*liking*” (i.e. higher thermometer scores). These are operationally identical because animosity refers to 100 minus one's thermometer score. Specifically, we use the difference between the true level of the (relevant) out-party rating (i.e. the actual average out-party thermometer score) and an individual's meta-perception estimate of that out-party rating. This difference provides a measure of the (in)accuracy of meta-perceptions. (We used poststratified weights in all cases.)

This is an ideal setting to test the possibility of static and dynamic effects as the two waves span a competitive event (4), and relevant intergroup context likely shifted around the election. This context enables us to explore static between-respondent differences and dynamic within-respondent changes and their relationships with meta-perception accuracy, respectively. We next discuss the explanatory variables on which we focus.

## Explanatory variables

While we did not have direct input on the items on the survey that generated the data, there were a number of relevant variables that might predict the accuracy of animosity meta-perceptions. As intimated, there is very little work that directly looks at predictors of partisan meta-perceptions generally or animosity meta-perceptions specifically. We selected predictor variables from the survey that extant work shows correlate with partisan animosity with the idea that factors that shape one's own out-party dislike may also affect what they think of the other party's dislike (i.e. animosity meta-perception).

Work on animosity suggests the following variables and relationships: meta-perception inaccuracy will increase with one's own dislike of the other party (and hence a negative relationship with like) (1, 3, 4, 25), conservativeness (due to decreased heterogenous contact) (26, 27), ideological extremity (6), lower network political diversity (28), higher levels of online political posting and/or higher levels of trust in social media (3, 29), and higher levels of trust in news media (30). These suggested relationships come from consideration of static comparisons, and thus, it is unclear whether they will hold dynamically^c^. One candidate for a predictor with distinct static and dynamic relationships to meta-perceptions is the frequency of political posting—both because one's own frequency can substantially change from *t*1 to *t*2 and because the information ecology to which one is exposed while posting can change. Other such candidates include the political diversity of one's friends and family network, trust in social media information, and trust in news media. These variables could be influenced by contextual changes but less so than political posting. In an electoral context, we expect greater change in how parties are discussed in political social media postings than in conversations within social networks (which do not necessitate political content). The same holds for social media trust and news media trust.

We acknowledge a limitation of our study is the absence of data on how the information context changed from time 1 to time 2. Some work suggests that leading up to an election, political partisan discussion is prevalent and negative, relative to after an election (31, 32). This suggests that the information ecology changes from more competitive (preelection) to relatively more cooperative (postelection). Yet, 2020 was far from typical given the losing candidate never conceded defeat. Our goal here is not to make definitive statements about acute relationships but rather to explore whether static and dynamic relationships among these variables differ; if so, it suggests a need to consider both in the study of political perceptions. Nor is our goal to make causal inferences about the “effect” of the election. Our goal is to descriptively demonstrate that temporal variability is a critical dimension of meta-perception accuracy that can reveal meaningfully different relationships with theorized predictors of (in)accuracy.

## Model framework: predicting changes in meta-perception using within–between panel models

To evaluate both static and dynamic relationships, we used within–between mixture models (33, 34), modeled via the *panelr* package (35) in R. All models used poststratification weighting. Within–between models disaggregate within- and between-person associations via a demeaning procedure to isolate (ⅰ) how change over time in predictors (e.g. change in social media postings from *T*1 to *T*2 relative to no change/less change/opposite direction change) is associated with change over time in the outcome of meta-perception inaccuracy (i.e. within-effects), from (ⅱ) the mean association between predictor (e.g. from a high value relative to a low value) and meta-perception accuracy outcome averaged across time (i.e. between-effects), while modeling random intercepts for participants. That is, we interpret time-invariant between-person predictors in terms of static relationships and changes in within-person variables as dynamic relationships. All variables modeled as within were also modeled as between, whereas between variables were not all modeled as within (i.e. control variables that are time-invariant over the period we examine).

This within–between model aligns with our framework because it can identify predictors of meta-perception inaccuracy change within a person, over time, while controlling for variables theoretically confounded with meta-perceptions, all within a unified hierarchical model. To be clear, in this framework, static relationships isolate overall inaccuracy/negativity, and dynamic relationships look at increases in inaccuracy pre- to postelection. This approach addresses the issues traditional cross-lagged panel analyses have in identifying within-person effects (36, 37). It also differs from fixed-effect (FE) panel analyses used in econometrics, which are designed to precisely isolate individual effects over time (within-effects) from all other variance, rather than model them simultaneously. Similar to the FE approach, within–between models isolate within-effects by demeaning time-varying predictors (FE models also demean the outcome variable). In this regard, within-person coefficients of within–between models are identical to the FE equivalent coefficient. In contrast to FE models, which then control for all other higher-level variables such as individuals (and anything else that structures the temporal nature of the data) using dummy variables, within–between models use random effects to model those structures hierarchically. They then add the predictor means (the value subtracted out in the “demeaning” of within-variables) into the model as between-effects (see (33) for discussion of fixed versus mixture model approaches to panel data).

## Descriptive changes in meta-perception

Our measure of inaccurate meta-perceptions began with the (weighted) mean thermometer rating for Democrats (toward Republicans) and for Republicans (toward Democrats), for Wave 1 (actual out-party liking: M_Dem_W1 = 18.92, M_Rep_W1 = 17.80) and Wave 2 (M_Dem_W2 = 22.24, M_Rep_W2 = 20.75). We then subtracted (from these respective true values) each participant's perceived (raw meta-perception) out-party attitude toward one's own party (e.g. Democrats’ beliefs about Republicans’ thermometer ratings toward Democrats). As such, meta-perception inaccuracy values greater than zero represent overestimating out-party negativity toward one's own party (e.g. if true out-party liking of one's own party was 20 and the participant [meta-]perceived it to be 5, we subtracted 5 from 20 to model meta-perception inaccuracy, with the resulting 15 representing a 15-point overestimation of out-party dislike). In short, a score above zero indicates negative inaccuracies (i.e. the respondent believed the other party dislikes their party more than they actually do) and a score below zero suggests positive inaccuracies (i.e. the respondent believed the other party likes their party more than they actually do).

**Fig. 1.**
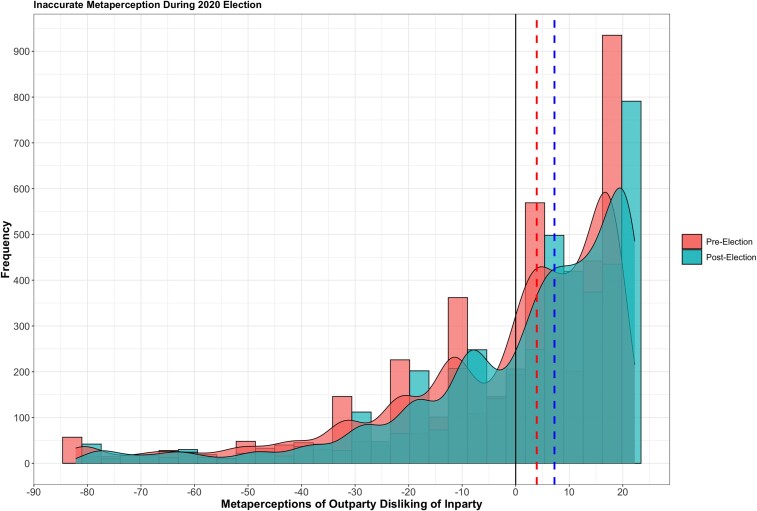
Histogram and density plot of meta-perception inaccuracy. Meta-perception inaccuracy is computed by (true like—perceived liking). Values greater than zero represent overestimating dislike. The dotted lines are medians by wave. Solid line is at zero, where judgments of out-party dislike of the in-party are accurate. *Y*-axis is observation frequency.

Figure 1 presents a visualization of the scores for Wave 1 and Wave 2. The figure clearly shows that the bulk of respondents displayed inaccuracy, such that they thought the other party disliked them more than they actually did. In Wave 1, 59.29% of responses were inaccurate and overly negative (inaccuracy > 0, MedianW1 = 3.92); however, at the sample average level, this was not statistically significantly different from zero in W1 (*V* = 4961741, *P* = 0.084): respondents were in fact accurate on average but the central tendency misses substantial variation in meta-perceptions. At Wave 2, 66.58% of responses were inaccurate and overly negative (inaccuracy > 0, MedianW2 = 7.24), and this was statistically significantly different from zero in W2 (*V* = 6025637, *P* < 0.001). Note that for the static analyses we computed the average of respondents’ inaccuracy at Wave 1 and Wave 2 to serve as the outcome variable, although the results are robust if we instead looked only at Wave 1 or Wave 2. All tests of mean differences used nonparametric tests to account for the high skew of the meta-perception data: a Wilcoxon rank sum test for independent samples and a Wilcoxon signed rank test for dependent samples. All tests were two-tailed.

Figure 1 shows an increase in inaccurate and overly negative meta-perceptions from Wave 1 (MedianW1 = 3.92) to Wave 2 (MedianW2 = 7.24, *V* = 3467320, *P* < 0.001). The aggregate increase in inaccuracy over time stemmed from both the raw meta-perceptions of out-party dislike becoming more negative postelection (a mean increase in meta-perception of disliking = 1.31, *V* = 3759262, *P* = 0.007), and actual out-party dislike decreasing (a mean decrease in actual disliking = −3.13, *V* = 2531483, *P* < 0.001). This is contrary to our (soft) expectation that meta-perceptions become more accurate (less negative) after a competitive election, although, again, 2020 was a unique year given the election results were not uniformly accepted. We probe dynamic relationships by looking at the associations between individuals’ changes in the variables discussed earlier and their meta-perception changes from Wave 1 to Wave 2 (e.g. did posting more on social media between pre- and postelection lead to less inaccurate meta-perceptions pre- versus postelection?)^d^.

## Static and dynamic relationships

We next turn to relationships, starting with the static relationships. We regressed inaccurate meta-perceptions (i.e. higher values indicate more inaccuracy) on each of the explanatory variables. We also controlled for partisanship, age, race, education, and income (we include the former two in the table, but for space/presentational reasons, we report results for the latter set in the supporting information)^e^. We display the results in Table 1. When interpreting the regression coefficients in Table 1, two important things are of note. First, the dependent variable is *overestimating* out-party hostility (i.e. negative and inaccurate animosity meta-perceptions). As such, a positive coefficient means that inaccuracy *increases* as the predictor increases, and vice versa. Second, all regression coefficients are standardized, reflecting changes in units of SDs. In Table 1, the within-person effects are consistently smaller than the between-person effects, which are themselves small in effect size (the largest being *ß* = 0.072). This reflects the fact that variance in between-person variables, by their nature, contains between-person confounds and the accumulation of past within-person effects over time, whereas the within-person variables as modeled isolate that variance and reflect only proximal changes between the two waves.

**Table 1.** Inaccurate meta-perceptions regressions.

Models predicting inaccurate meta-perceptions		
	Between-only model	Between–within model
Out-party liking (between)	−0.235 (−0.258, −0.212) (*P* < 0.000)	−0.257 (−0.284, −0.229) (*P* < 0.000)
Out-party liking (within)		−0.072 (−0.087, −0.056) (*P* < 0.000)
Conservatism (between)	0.082 (0.047, 0.117) (*P* < 0.000)	0.054 (0.013, 0.095) (*P* = 0.010)
Conservatism (within)		0.034 (0.019, 0.049) (*P* < 0.000)
Ideological extremism (between)	0.052 (0.028, 0.076) (*P* < 0.000)	0.028 (0.001, 0.055) (*P* = 0.042)
Ideological extremism (within)		0.027 (0.012, 0.042) (*P* < 0.001)
Network diversity (between)	0.037 (0.015, 0.059) (*P* = 0.001)	0.071 (0.045, 0.097) (*P* < 0.000)
Network diversity (within)		−0.011 (−0.026, 0.005) (*P* = 0.178)
Political posting online (between)	0.006 (−0.017, 0.030) (*P* = 0.608)	0.018 (−0.008, 0.044) (*P* = 0.165)
Political posting online (within)		−0.015 (−0.031, 0.001) (*P* = 0.064)
Social media info. trust (between)	−0.140 (−0.163, −0.118) (*P* < 0.000)	−0.156 (−0.183, −0.129) (*P* < 0.000)
Social media info. trust (within)		−0.041 (−0.057, −0.026) (*P* < 0.000)
News media info. trust (between)	−0.008 (−0.038, 0.021) (*P* = 0.575)	−0.006 (−0.041, 0.029) (*P* = 0.734)
News media info. trust (within)		−0.005 (−0.021, 0.011) (*P* = 0.565)
Party ID-Republican	−0.380 (−0.459, −0.301) (*P* < 0.000)	−0.325 (−0.411, −0.240) (*P* < 0.000)
Age	−0.097 (−0.123, −0.071) (*P* < 0.000)	−0.098 (−0.124, −0.072) (*P* < 0.000)
*N*	8,622	8,622
*N* (case id)	4,311	4,311
Akaike information criterion (AIC)	24,992.298	24,986.211
Bayesian information criterion (BIC)	25,246.533	25,289.880
*R* ^2^ (fixed)	0.135	0.153
*R* ^2^ (total)	0.432	0.444

Predicting inaccuracy, so positive coefficient means more inaccurate, and vice versa. All models control for Race, Education, and Income and use poststratification weighting. All estimates are standardized. Parentheses are 95% CIs. *P*-values calculated using Welsh–Satterwhite d.f. approximation. Full model significantly improved model fit, *X*^2^(7) = 73.29, *P* < 0.001.

We found that out-party disliking, ideological extremism, and conservatism were all associated with increased inaccuracy. Contrary to what some prior work suggests (e.g. (28)), increased network diversity was associated with more inaccuracy. It could be that those with more diverse networks avoid political discussions (38) that could otherwise counter exaggerated stereotypes. Alternatively, it could reflect a type of backfire effect (39) due to highly visible opposing partisans who are particularly hostile (40). Also, of particular note, was the absence of a relationship between self-reported political posting on social media and the accuracy of meta-perceptions. However, this null effect accords with Nyhan et al.'s (41) finding that deactivating access to Facebook prior to the 2020 election had no influence on affective polarization. Moreover, having more trust in social media information was associated with more accurate perceptions. This may reflect that those who are more trusting of social media information tend to maintain higher levels of empathy and goodwill (42) which, in turn, reduces negative meta-perceptions. Interestingly, we observe no association with trust in news media, either between- or within-persons, and accuracy. Regardless, the results starkly contrasted with caricatures of social media driving negative misperceptions (3, 29). Finally, we found partisanship displayed the inverse impact of ideology such that Republicans demonstrated less inaccuracy and older individuals also displayed less inaccuracy. The former result could stem from a partisan difference, an incumbency difference, or a candidate difference (e.g. Trump versus Biden). The latter result is sensible given younger people, today, exhibit (relatively) greater animosity (43) and thus likely impute animus to the other party.

We next turn to the relationship between the change in meta-perception inaccuracy and change in each variable—that is, looking at how these variables operated in dynamic relationships—while retaining between-respondent predictor variables^f^. Out-party liking, ideological extremism, and conservatism exhibited the same relationships between (statically) and within (dynamically) partisans. For example, partisans with more affinity for the other party possessed more accurate meta-perceptions; analogously, as a given partisan increased their affinity from pre- to postelection, their meta-perceptions became more accurate. We also found consistency with social media trust: relatively trusting partisans were more accurate and those who became more trusting increased in their accuracy.

The other variables, however, offered a different picture. The surprising between-respondent network diversity result remained significant once we included the within predictors, but within-respondent, it showed no relationship: changes in meta-perceptions did not track with changes in network diversity (although this could reflect insufficient statistical power). Moreover, whereas those who posted online about politics relatively more did not significantly differ in their meta-perceptions accuracy from those who posted less in the between analysis, an *increase* in posting by an individual, from pre- to postelection was associated with *more accurate* meta-perceptions. This is a vital result for two reasons. First, neither this nor the (negative) trust in social media information finding are consistent with common assertions that engagement on political social media contributes to exaggerated meta-perceptions (29, 44). Second, inferences between individuals about a relationship between variables (static) can differ from inferences about change within an individual (dynamic).

As we discussed, we suspect this reflects variation in context—inevitably the nature of social media content changes over-time. Interestingly, Facebook users in the sample reported political discourse becoming *more* negative postelection, M_W1_ = 2.05, M_W2_ = 1.97, *t*(3,732) = 4.95, *P* < 0.001, *d*_paired_ = 0.08). This may reflect the contentious postelection environment, with wide-ranging claims of fraud (and is somewhat consistent with the increased negativity in meta-perceptions we report in Fig. 1). It also contrasts with the idea (we discussed earlier) that increased meta-perception accuracy from posting more over-time reflected an, on average, improvement in the quality/positivity of the social media environment. The exact dynamic at work is not clear; for instance, it could be that this was unique to Facebook (the question about perceived political discourse quality specifically asked about Facebook only; therefore, respondents represent only a subset of the sample), and/or it could be reflective of substantial heterogeneity across the social media environment and/or perceptions thereof (and how one selects into posting more/less). Regardless, the distinct between- and within-person results make clear that statements of “social media effects” need to be explicit about the relevant counterfactual, such as “effects” between individuals during a given time period or “effects” on an individual who changes consumption.

In sum, the between-only (static) results cohere with expectations when it comes to out-party (dis)liking, conservatism, and ideological extremity. We find the inverse of what we expected for network diversity and trust in social media information and null results for posting political content on social media and trust in news media. Importantly, it is the variables that involve the communication environment that run counter to the expectations we previously discussed. These variables are presumably more difficult to predict because they depend on context—i.e. the information environment. Moreover, as expected, the within (dynamic) relationships are weaker than the between (dynamic) relationships (comparing coefficients) and can show very different relationship patterns, particularly with communication-oriented variables. This may reflect a mixture of the aforementioned differences in variance as well as the possibility that dynamic relationships are nosier.

## Conclusion

The study of misperceptions including meta-perceptions has become central to the study of political polarization. Many point to them as a threat to social and democratic functioning, suggesting that correcting them will bring salubrious outcomes. Indeed, Ruggeri et al. (12), p. 1377, suggest that such corrections have “the potential to increase social cohesion and wellbeing of populations around the world” (see also (7)). Ironically, employing and testing such interventions—that provide correct information about what opposing partisans believe (e.g. (1, 4))—have outpaced an understanding of who exactly holds exaggerated meta-perceptions. Such knowledge not only provides insight into the origins of inaccurate meta-perceptions, but also offers applied data on how to target interventions. This is valuable, particularly given recent work that suggests corrections with accurate information (e.g. out-partisans do not harbor extreme animus) are not robust (45) and that meta-perceptions are unstable in general (46). Our findings showed that the lack of over-time stability does not inherently indicate nonsystematic changes, but rather forces that lie at the intersection of individual attributes and contextual circumstances. Moreover, our results offer an initial foray into predictors which, in turn, could allow for more targeted corrections. Nearly all the work on corrections focuses on general rather than targeted messages, even though a large literature shows targeted political communications can be effective (47) as can engagement with particular individuals who share one's interests and/or characteristics (48). Crafting matched interventions could be a worthwhile avenue to pursue given the inherent challenges in creating enduring interventions due to the stickiness of polarized norms (49). In exploring targeted interventions, though, it is important to differentiate their potential effects on beliefs, behaviors, and attitudes (10) and recognize that a priori forecasting the success of any intervention is a challenge (50). We do not mean to caste too much pessimism regarding corrections, however; indeed, the fact that corrections have been shown to work at all suggests meta-perceptions reflect more than ingrained out-party stereotypes and the systematic changes in meta-perceptions we observe here make clear that they are responsive to various informational cues.

We acknowledge the limited generalizability of our findings; the 2020 US election was unlike any that preceded it (e.g. (44, 51)). Yet, one of our points is that generalizing any relationship is challenging because it requires sensitivity to context and time. While this is not a novel insight (work on external validity has long recognized it; e.g. (19, 52)), it is one that receives little attention in practice (although see (53, 54)). Ours is a first-order demonstration of Lees and Cikara's (4) observation that distinct contexts shape meta-perceptions. Going forward, identifying context sensitivity while also controlling for individual differences requires the study of within-person changes over time in response to specific external event (i.e. the 2020 election). This approach also facilitates causal inference, particularly with nonexperimental data.

Our most intriguing substantive finding concerns social media. Existing research on social media and polarization nearly always poses the question of whether social media contributes to various types of polarization or whether there are no effects (c.f. (29, 39, 41, 55–58)). Our dynamic relationship results reveal that in certain situations, social media engagement can depolarize. This presumably occurs because the context changed. One could infer from the results that the postelection environment became more cooperative (e.g. (59)) and less attack oriented (23). Yet, the fact that Facebook users reported *more* negativity in political discussions on Facebook postelection suggests that any one platform may not be representative of the broader social media environment and/or that the average perception of discussions are heterogeneous and/or inaccurate. Clearly, the next iteration on work about within-person change should directly measure (perceptions of) the communication context.

Along these lines, dynamic relationships are most likely to differ from static ones when the variables involved are influenced by the information context (e.g. whether messaging is more competitive or cooperative). This, in turn, is more likely to influence communication-oriented variables (e.g. social media posting, online social networks) that, by definition, entail consumption of content from that context. Future work should move beyond what we did by explicitly theorizing and, as mentioned, measuring variations in context. This is a challenging task, at least with regard to social media, given proprietary data. As González-Bailón and Lelkes (56: 165) explain, “More research is necessary to uncover…causal connections, and this will likely require collaboration with social media platforms as well as more inclusive policies for data access.”

Methodologically, our findings suggest that research interested in the origins and nature of meta-perceptions needs to address four questions. First, what time period is being studied, and how does the nature of the context compare to other periods? Second, what predictor variables are relevant—these might include individual variables such as ideological extremity or situational variables such as the cooperative/competitive nature of the context (which themselves may interact with distinct contextual factors)? Most work, including ours, only measures individual and not contextual variables. Doing both would facilitate the study of the intersection between level of measurement (individual, contextual) and type of relationship (static, dynamic). Third, do relevant predictor variables change over the period being studied? Fourth, if so, what inference is most relevant—studying differences between types of individuals or studying the changes an individual experiences? There is not a “correct” answer. The crucial insight is that any inference needs to explicitly clarify the times to which they apply and whether they concern static differences or dynamic change. Given the crucial role that meta-perceptions ostensibly play in politics, and the distinct long-standing debates on the role of social media in society, it is crucial that researchers refrain from over-generalizing and consider the temporal limitations and realities of any inference.

## Materials and methods

### Participants

Data were drawn from the American National Election Studies 2020 Social Media Study (https://electionstudies.org/data-center/2020-social-media-study/), a two-wave online panel study conducted before (August 20–September 17) and after (November 9–January 1, 2021) the 2020 US presidential election. Ethical approval for this data collection was provided by Stanford University (eProtocol #57100). A total of 5,277 participants completed the survey at both waves. We removed two participants who did not respond to the party identification question at Wave 2, and 462 participants whose party leaning changed from Wave 1 and Wave 2 (44). For these switchers, the outcome variables would be inconsistent since they would be answering thermometer questions about different parties in each wave. We also removed 364 true independents (24) and 60 participants who did not respond to the primary dependent variable at both waves (perceptions of out-party liking of the in-party), leaving a final *N* = 4,389. All models used pairwise deletion of incomplete data, hence an *N* = 4,311 in the final reported model. In the final sample, the average age was 51.92 (SD = 16.72), 55.59% identified as Democrat and 44.41% as Republican, 71.93% identified as White, 10.12% as Black, 11.41% as Hispanic, and 6.54% as “Other.” 2.87% had less than a high school degree, 13.24% completed high school, 37.5% completed “some” college or an associates, 26.45% completed a bachelors, and 19.94% completed a graduate degree.

### Variables

Meta-perception inaccuracy was measured by subtracting responses to the question “How do you think the typical [out-party] voter would rate the [in-party]” on a thermometer scale from the poststratification weighted mean thermometer rating (0–100) by party (the true values). Participants’ out-party liking was their thermometer rating of their out-party. Conservatism was measured using a 1–7 Likert measure of “Very liberal” (1) to “Very conservative (7). Ideological extremity was measured as the absolute value of the liberalism-conservatism measure after it was transformed to a −3 to +3 scaling. Network political diversity was measured with the question “How many of your family and friends are [out-partisans]” on a 1–5 Likert scale from “None or almost none” (1) to “All or nearly all” (5). Online political posting frequency was measured by asking “During the past 12 months, how often did you do each of the following” regarding “wrote and posted political messages online” on 1–5 Likert scales from “Never” (1) to “A lot” (5). Trust in social media information was measured as the average of two items asking “How much do you think political information from each of these sources [Facebook posts/Twitter posts] can be trusted” on 1–5 Likert scales from “Not at all” (1) to “A great deal” (5). Trust in news media was measured as the average of three items asking “How much do you think political information from each of these sources [MSNBC/New York Times/USA Today] can be trusted” on 1–5 Likert scales from “Not at all” (1) to “A great deal” (5). The analyses were not preregistered, we did not conduct an a priori power, and we were not directly involved in the data collection. The means and SDs for all variables are given in Table S5.

## Supplementary Material

pgae324_Supplementary_Data

## Data Availability

All analyses reported herein will be made available publicly on the Open Science Framework upon publication of this work in a peer-reviewed journal (link to analyses for peer-reviewers: https://osf.io/unmvs/?view_only=2df21c718e094b8db2b23b22fc36aab6).

## Notes

a This distinction is addressed in experimental research with random assignment.

b Another reason for stronger static relationships is that there is a greater risk of omitted variable bias in the static analyses since, as mentioned, the dynamic analyses can control for time-invariant unobserved variables.

c In the supporting information, we explore three other variables (political knowledge, online network diversity, and perceived social media discourse quality). We did not include them in the main analyses because they were either measured only at one point in time (and thus dynamic relationships cannot be studied) or measured only among a subset of respondents. The inclusion of these new variables never meaningfully changes the findings reported here.

d While not shown in the figure, we find that, at wave 1, Democrats were more inaccurate and negative than Republicans (*W* = 2,490,650, *P* = 0.007), but at wave 2 there was no difference in the parties’ levels of inaccuracy (*W* = 2,359,269, *P* = 0.657).

e Specifically, the age, race, education, and income variables are measured categorically and thus make the results table visually difficult when added. The SEs are clustered within-respondent.

f We do not look at within changes in age or partisanship as these are highly stable variables in the time period we study. (Ideology, in contrast, demonstrated a fair amount of change with 36.2% of the sample changing their ideology between periods.)
