# Supplementary material for: Why partisans feel hated: Distinct static and dynamic relationships with animosity meta-perceptions
Source: PNAS Nexus. 2024 Oct 15;3(10):pgae324. doi: 10.1093/pnasnexus/pgae324 (PMC11475464; doi:10.1093/pnasnexus/pgae324)
Supplement: pgae324_Supplementary_Data [file pgae324_supplementary_data.docx]

**Supplemental Materials for**

***Why Partisans Feel Hated: Distinct Static and Dynamic Relationships with Animosity Meta-Perceptions***

**Supplemental Table 1a: Between Only Model Fully Displayed**

Supplemental Tables 1a and 1b are the full regression outputs for the models reported in Table 1 of the primary manuscript. In Table 1 we removed output related to Race, Income, and Education, because they served as control variables and including them would have been visually disruptive.

Note that the Race, Income, and Education variables are dummy coded relative to the lowest level for Income (<$5k) and Education (Less than High School), and relative to White for Race.

|  | Between-Only Model |
| --- | --- |
| (Intercept) | -0.521 [-0.735, -0.306] (p < 0.000) |
| Outparty Liking (Between) | -0.235 [-0.258, -0.212] (p < 0.000) |
| Social Media Info Trust (Between) | -0.140 [-0.163, -0.118] (p < 0.000) |
| Network Diversity (Between) | 0.037 [0.015, 0.059] (p = 0.001) |
| Political Posting Online (Between) | 0.006 [-0.017, 0.030] (p = 0.608) |
| Ideological Extremism (Between) | 0.052 [0.028, 0.076] (p < 0.000) |
| Conservatism (Between) | 0.082 [0.047, 0.117] (p < 0.000) |
| News Media Info Trust (Between) | -0.008 [-0.038, 0.021] (p = 0.575) |
| Age | -0.097 [-0.123, -0.071] (p < 0.000) |
| Party ID-GOP | -0.380 [-0.459, -0.301] (p < 0.000) |
| RaceBlack | -0.231 [-0.319, -0.143] (p < 0.000) |
| RaceHispanic | -0.117 [-0.199, -0.035] (p = 0.005) |
| RaceOther | -0.105 [-0.206, -0.004] (p = 0.041) |
| EducationGrad | 0.109 [-0.028, 0.246] (p = 0.120) |
| EducationBA | 0.115 [-0.016, 0.246] (p = 0.087) |
| EducationHS | 0.004 [-0.128, 0.136] (p = 0.949) |
| EducationSome_College_Asso | 0.046 [-0.080, 0.172] (p = 0.472) |
| `Income$85k` | 0.678 [0.480, 0.876] (p < 0.000) |
| `Income$40k` | 0.636 [0.436, 0.836] (p < 0.000) |
| `Income$20k` | 0.687 [0.473, 0.900] (p < 0.000) |
| `Income$75k` | 0.717 [0.505, 0.930] (p < 0.000) |
| `Income$175k` | 0.684 [0.438, 0.931] (p < 0.000) |
| `Income$100k` | 0.698 [0.499, 0.898] (p < 0.000) |
| `Income$10K` | 0.835 [0.608, 1.062] (p < 0.000) |
| `Income$150k` | 0.637 [0.411, 0.863] (p < 0.000) |
| `Income$125k` | 0.737 [0.528, 0.946] (p < 0.000) |
| `Income$50k` | 0.681 [0.480, 0.881] (p < 0.000) |
| `Income$60k` | 0.705 [0.509, 0.901] (p < 0.000) |
| `Income$35k` | 0.575 [0.360, 0.791] (p < 0.000) |
| `Income$25k` | 0.654 [0.442, 0.866] (p < 0.000) |
| `Income$30k` | 0.614 [0.403, 0.825] (p < 0.000) |
| `Income$15k` | 0.545 [0.324, 0.766] (p < 0.000) |
| `Income$200k+` | 0.775 [0.549, 1.001] (p < 0.000) |
| `Income$5K` | 0.538 [0.287, 0.789] (p < 0.000) |
| N | 8622 |
| N (caseid) | 4311 |
| AIC | 24992.298 |
| BIC | 25246.533 |
| R2 (fixed) | 0.135 |
| R2 (total) | 0.432 |
| Note: Predicting inaccuracy, so positive coefficient means more inaccurate, and vice versa. All models use post-stratification weighting. All estimates are standardized. Brackets are 95% confidence intervals. p-values calculated using Welsh-Satterwhite d.f. approximation. Full model significantly improved model fit, X2(7) = 73.29, p < 0.001. | |

**Supplemental Table 1b: Within-Between Model Fully Displayed**

|  | Between-Within Model |
| --- | --- |
| (Intercept) | -0.506 [-0.720, -0.292] (p < 0.000) |
| Outparty Liking (Between) | -0.257 [-0.284, -0.229] (p < 0.000) |
| Social Media Info Trust (Between) | -0.156 [-0.183, -0.129] (p < 0.000) |
| Network Diversity (Between) | 0.071 [0.045, 0.097] (p < 0.000) |
| Political Posting Online (Between) | 0.018 [-0.008, 0.044] (p = 0.165) |
| Ideological Extremism (Between) | 0.028 [0.001, 0.055] (p = 0.042) |
| Conservatism (Between) | 0.054 [0.013, 0.095] (p = 0.010) |
| News Media Info Trust (Between) | -0.006 [-0.041, 0.029] (p = 0.734) |
| Outparty Liking (Within) | -0.072 [-0.087, -0.056] (p < 0.000) |
| Social Media Info Trust (Within) | -0.041 [-0.057, -0.026] (p < 0.000) |
| Network Diversity (Within) | -0.011 [-0.026, 0.005] (p = 0.178) |
| Political Posting Online (Within) | -0.015 [-0.031, 0.001] (p = 0.064) |
| Ideological Extremism (Within) | 0.027 [0.012, 0.042] (p = 0.001) |
| Conservatism (Within) | 0.034 [0.019, 0.049] (p < 0.000) |
| News Media Info Trust (Within) | -0.005 [-0.021, 0.011] (p = 0.565) |
| Age | -0.098 [-0.124, -0.072] (p < 0.000) |
| Party ID-GOP | -0.325 [-0.411, -0.240] (p < 0.000) |
| RaceBlack | -0.168 [-0.258, -0.079] (p < 0.000) |
| RaceHispanic | -0.100 [-0.181, -0.018] (p = 0.016) |
| RaceOther | -0.084 [-0.185, 0.017] (p = 0.104) |
| EducationGrad | 0.091 [-0.045, 0.228] (p = 0.191) |
| EducationBA | 0.096 [-0.035, 0.227] (p = 0.150) |
| EducationHS | -0.004 [-0.136, 0.127] (p = 0.949) |
| EducationSome_College_Asso | 0.030 [-0.095, 0.156] (p = 0.638) |
| `Income$85k` | 0.635 [0.438, 0.833] (p < 0.000) |
| `Income$40k` | 0.605 [0.406, 0.805] (p < 0.000) |
| `Income$20k` | 0.665 [0.452, 0.879] (p < 0.000) |
| `Income$75k` | 0.680 [0.467, 0.892] (p < 0.000) |
| `Income$175k` | 0.654 [0.408, 0.899] (p < 0.000) |
| `Income$100k` | 0.658 [0.459, 0.857] (p < 0.000) |
| `Income$10K` | 0.825 [0.599, 1.052] (p < 0.000) |
| `Income$150k` | 0.605 [0.380, 0.831] (p < 0.000) |
| `Income$125k` | 0.692 [0.484, 0.901] (p < 0.000) |
| `Income$50k` | 0.642 [0.442, 0.842] (p < 0.000) |
| `Income$60k` | 0.671 [0.476, 0.867] (p < 0.000) |
| `Income$35k` | 0.553 [0.338, 0.768] (p < 0.000) |
| `Income$25k` | 0.630 [0.419, 0.842] (p < 0.000) |
| `Income$30k` | 0.585 [0.375, 0.796] (p < 0.000) |
| `Income$15k` | 0.508 [0.287, 0.728] (p < 0.000) |
| `Income$200k+` | 0.731 [0.506, 0.957] (p < 0.000) |
| `Income$5K` | 0.523 [0.273, 0.773] (p < 0.000) |
| N | 8622 |
| N (caseid) | 4311 |
| AIC | 24986.211 |
| BIC | 25289.880 |
| R2 (fixed) | 0.153 |
| R2 (total) | 0.444 |
| Note: Predicting inaccuracy, so positive coefficient means more inaccurate, and vice versa. All models use post-stratification weighting. All estimates are standardized. Brackets are 95% confidence intervals. p-values calculated using Welsh-Satterwhite d.f. approximation. Full model significantly improved model fit, X2(7) = 73.29, p < 0.001. | |

**Supplemental Table 2: Online Network Diversity added to Within-Between Model**

Below is the full within-between model reported in the manuscript, with the addition of a self-reported measure of online social network (political) diversity as a within- and between-persons predictor. Like the personal network diversity measure, it is coded for the participant’s outparty (e.g., if you’re Democrats, how many Republicans friends do you have online, and vice versa). The online network diversity item was only administered to participants with active Facebook accounts, hence the significant drop in sample size from the models in the main text (N = 4311 to N = 3013) via pairwise deletion.

|  | Between-Within Model |
| --- | --- |
| Outparty Liking (Between) | -0.257 [-0.290, -0.225] (p < 0.000) |
| Conservatism (Between) | 0.057 [0.011, 0.104] (p = 0.016) |
| Ideological Extremism (Between) | 0.036 [0.005, 0.068] (p = 0.024) |
| Network Diversity (Between) | 0.022 [-0.015, 0.060] (p = 0.247) |
| Political Posting Online (Between) | 0.018 [-0.013, 0.049] (p = 0.251) |
| Social Media Info Trust (Between) | -0.170 [-0.202, -0.138] (p < 0.000) |
| News Media Info Trust (Between) | -0.009 [-0.049, 0.031] (p = 0.662) |
| Online Network Diversity (Between) | 0.074 [0.037, 0.112] (p < 0.000) |
| Party ID-GOP | -0.375 [-0.472, -0.277] (p < 0.000) |
| Age | -0.083 [-0.113, -0.053] (p < 0.000) |
| Outparty Liking (Within) | -0.073 [-0.092, -0.054] (p < 0.000) |
| Conservatism (Within) | 0.036 [0.018, 0.055] (p < 0.000) |
| Ideological Extremism (Within) | 0.029 [0.010, 0.047] (p = 0.002) |
| Network Diversity (Within) | -0.018 [-0.037, 0.001] (p = 0.070) |
| Political Posting Online (Within) | -0.012 [-0.031, 0.008] (p = 0.245) |
| Social Media Info Trust (Within) | -0.043 [-0.062, -0.024] (p < 0.000) |
| News Media Info Trust (Within) | -0.012 [-0.032, 0.007] (p = 0.208) |
| Online Network Diversity (Within) | 0.016 [-0.004, 0.035] (p = 0.116) |
| N | 6026 |
| N (caseid) | 3013 |
| AIC | 17173.403 |
| BIC | 17475.076 |
| R2 (fixed) | 0.154 |
| R2 (total) | 0.406 |
| Note: Predicting inaccuracy, so positive coefficient means more inaccurate, and vice versa. The model control for Race, Education, and Income, and uses post-stratification weighting. All estimates are standardized. Brackets are 95% confidence intervals. p-values calculated using Welsh-Satterwhite d.f. approximation. | |

**Supplemental Table 3: Political Knowledge added to Within-Between Model**

Below is the full within-between model reported in the manuscript, with the addition of an objective political knowledge question (correctly identifying Speaker Nancy Pelosi and Chief Justice Roberts), as a between-persons predictor. This item was only administered at Wave 2, and hence can only be modeled as between-persons.

|  | Between-Within Model |
| --- | --- |
| Outparty Liking (Between) | -0.250 [-0.278, -0.222] (p < 0.000) |
| Conservatism (Between) | 0.053 [0.012, 0.093] (p = 0.012) |
| Ideological Extremism (Between) | 0.021 [-0.006, 0.049] (p = 0.123) |
| Network Diversity (Between) | 0.069 [0.043, 0.094] (p < 0.000) |
| Political Posting Online (Between) | 0.014 [-0.012, 0.040] (p = 0.289) |
| Social Media Info Trust (Between) | -0.149 [-0.176, -0.122] (p < 0.000) |
| News Media Info Trust (Between) | -0.013 [-0.049, 0.022] (p = 0.453) |
| Political Knowledge (Between) | 0.061 [0.037, 0.086] (p < 0.000) |
| Party ID-GOP | -0.323 [-0.409, -0.238] (p < 0.000) |
| Age | -0.111 [-0.138, -0.084] (p < 0.000) |
| Outparty Liking (Within) | -0.073 [-0.088, -0.057] (p < 0.000) |
| Conservatism (Within) | 0.035 [0.019, 0.050] (p < 0.000) |
| Ideological Extremism (Within) | 0.027 [0.011, 0.042] (p = 0.001) |
| Network Diversity (Within) | -0.012 [-0.027, 0.004] (p = 0.150) |
| Political Posting Online (Within) | -0.016 [-0.032, -0.000] (p = 0.049) |
| Social Media Info Trust (Within) | -0.041 [-0.057, -0.026] (p < 0.000) |
| News Media Info Trust (Within) | -0.005 [-0.021, 0.011] (p = 0.554) |
| N | 8622 |
| N (caseid) | 4311 |
| AIC | 24971.606 |
| BIC | 25282.337 |
| R2 (fixed) | 0.156 |
| R2 (total) | 0.444 |
| Note: Predicting inaccuracy, so positive coefficient means more inaccurate, and vice versa. The model control for Race, Education, and Income, and uses post-stratification weighting. All estimates are standardized. Brackets are 95% confidence intervals. p-values calculated using Welsh-Satterwhite d.f. approximation. | |

**Supplemental Table 4: Social Media Discourse Quality added to Within-Between Model**

Below is the full within-between model reported in the manuscript, with the addition of a self-reported social media (political) discourse quality (perception that Facebook political conversations are “respectful” and “informative”) as a within- and between-persons predictor. The social media discourse quality items were only administered to participants with active Facebook accounts, hence the significant drop in sample size from the models in the main text (N = 4311 to N = 3021) via pairwise deletion.

|  | Between-Within Model |
| --- | --- |
| Outparty Liking (Between) | -0.248 [-0.280, -0.215] (p < 0.000) |
| Conservatism (Between) | 0.064 [0.018, 0.111] (p = 0.007) |
| Ideological Extremism (Between) | 0.034 [0.003, 0.066] (p = 0.032) |
| Network Diversity (Between) | 0.055 [0.024, 0.085] (p < 0.000) |
| Political Posting Online (Between) | 0.026 [-0.005, 0.056] (p = 0.103) |
| Social Media Info Trust (Between) | -0.120 [-0.156, -0.085] (p < 0.000) |
| News Media Info Trust (Between) | -0.005 [-0.045, 0.034] (p = 0.800) |
| Social Media Discourse Quality (Between) | -0.106 [-0.140, -0.072] (p < 0.000) |
| Party ID-GOP | -0.356 [-0.453, -0.259] (p < 0.000) |
| Age | -0.078 [-0.108, -0.048] (p < 0.000) |
| Outparty Liking (Within) | -0.072 [-0.091, -0.053] (p < 0.000) |
| Conservatism (Within) | 0.034 [0.016, 0.053] (p < 0.000) |
| Ideological Extremism (Within) | 0.031 [0.012, 0.049] (p = 0.001) |
| Network Diversity (Within) | -0.015 [-0.034, 0.004] (p = 0.112) |
| Political Posting Online (Within) | -0.011 [-0.031, 0.009] (p = 0.275) |
| Social Media Info Trust (Within) | -0.039 [-0.058, -0.020] (p < 0.000) |
| News Media Info Trust (Within) | -0.011 [-0.030, 0.008] (p = 0.262) |
| Social Media Discourse Quality (Within) | -0.023 [-0.042, -0.004] (p = 0.018) |
| N | 6042 |
| N (caseid) | 3021 |
| AIC | 17187.200 |
| BIC | 17488.992 |
| R2 (fixed) | 0.159 |
| R2 (total) | 0.409 |
| Note: Predicting inaccuracy, so positive coefficient means more inaccurate, and vice versa. The model control for Race, Education, and Income, and uses post-stratification weighting. All estimates are standardized. Brackets are 95% confidence intervals. p-values calculated using Welsh-Satterwhite d.f. approximation. | |

**Supplemental Table 5: Primary Variable Means and SDs Across Waves**

Below are the means and standard deviations for all the within-person variables, by wave. Descriptions of these items can be found in the “Variables” subsection in the Method section of the main text.

| **Variable** | **Wave 1 Mean (SD)** | **Wave 2 Mean (SD)** |
| --- | --- | --- |
| Meta-Perception Accuracy | -2.85 (22.6) | 1.57 (21.01) |
| Outparty Liking | 18.09 (19.73) | 21.23 (20.36) |
| Social Media Info Trust | 1.55 (0.7) | 1.49 (0.67) |
| Network Diversity | 2.35 (0.86) | 2.35 (0.86) |
| Ideological Extremism | 1.58 (1.04) | 1.63 (1.02) |
| Conservatism | 4.07 (1.89) | 4.04 (1.92) |
| Political Posting Online | 1.76 (1.21) | 1.75 (1.21) |
| News Media Info Trust | 2.5 (1.02) | 2.59 (1.08) |
